# Supplementary material for: COVID-19 Vaccination Is Associated with a Better Outcome in Acute Ischemic Stroke Patients: A Retrospective Observational Study
Source: J Clin Med. 2022 Nov 22;11(23):6878. doi: 10.3390/jcm11236878 (PMC9737827; doi:10.3390/jcm11236878)
Supplement: Supplementary file 1 [file jcm-11-06878-s001.zip › jcm-2040813-supplementary.pdf]

Supplementary Table S1. Multivariate Logistic Regression Analysis for Predicting outcome in stroke patients.

	OR	Lower 95% CI	Upper 95% CI	*p*-Value
Stroke Age > 65 yrs	1.879	0.867	4.075	0.110
Stroke Age > 85 yrs	**2.374**	**1.085**	**5.193**	**0.030**
NIHSS > 4 at stroke onset	1.460	0.687	3.101	0.325
NIHSS > 4 after discharge	**7.524**	**3.552**	**15.938**	**< 0.001**
Vaccine before stroke	**0.400**	**0.216**	**0.741**	**0.004**
Hypertension	2.313	0.839	6.378	0.105
Obesity	4.647	1.016	21.253	0.059
Previous stroke	**2.451**	**1.056**	**5.689**	**0.037**
Patent foramen ovale	0.000	0.000		0.999
Hospitalization for COVID-19	4.546	0.649	31.851	0.108
Intravenous thrombolysis	0.472	0.194	1.149	0.098
Endovascular mechanical thrombectomy	0.953	0.428	2.122	0.906
Coagulopathy	0.551	0.112	2.725	0.465
COPD	1.701	0.594	4.875	0.127

NIHSS: National Institute of Health Stroke Scale; COPD: Chronic obstructive pulmonary disease. Values which resulted statistically significant in the univariate analysis are highlighted in bold character.
